# Perceived Use of Web-Based Videoconferencing for Social Connectedness Among Older Adults Living in Long-Term Care: Qualitative Study

**DOI:** 10.2196/73213

**Published:** 2026-01-09

**Authors:** Anna Garnett, Halyna Yurkiv, Denise M Connelly, Richard Booth, Lorie Donelle

**Affiliations:** 1 Arthur Labatt Family School of Nursing, Western University, 1151 Richmond Street, London, ON, N6A 3K7, Canada, +1 519-661-2111; 2 School of Physiotherapy, Western University, London, ON, Canada; 3 Biobehavioral Health & Nursing Science, College of Nursing, University of South Carolina, South Carolina, SC, United States

**Keywords:** web-based, videoconferencing, virtual technology, long-term care, social connectedness, older adults

## Abstract

**Background:**

The COVID-19 pandemic highlighted how restrictions on in-person interactions within long-term care homes (LTCHs) severely compromised social connectedness among older adults and their families. Post pandemic, despite policy changes supporting greater in-person family engagement, frequent outbreaks continue to disrupt face-to-face interactions, and factors such as geography, life circumstances, and health can constrain family members’ ability to make regular in-person visits. Research suggests that web-based videoconferencing technology (WVT) may be a practical solution to help older adults within LTCHs to maintain social connection in the absence of physical gathering. However, increased understanding of end user experience is lacking, and more information on LTCHs’ readiness to support and sustain WVT will be needed if this modality is to be successfully and widely implemented.

**Objective:**

This study aimed to understand how older adults living in LTCHs, their families, and LTCH staff members perceived the use and ease of use of WVT devices for facilitating social connectedness.

**Methods:**

Using a qualitative description approach, in-depth semistructured interviews were conducted with 7 older adults, 22 family members, and 10 staff across 3 LTCHs via Zoom (Zoom Communications, Inc), Microsoft Teams, or phone calls. Data were analyzed using a directed content analysis informed by the technology acceptance model.

**Results:**

Findings were structured into 3 main themes: actual system use, perceived usefulness of WVT, and perceived ease of use of WVT. Participants described using a range of WVT hardware and software to promote social connection between older adults and family members. Videoconferencing had a crucial role in supporting older adults and their family members’ positive emotional state while also enabling them to maintain life and social roles such as participating in family functions. Despite the perceived use of these tools, participants were concerned about the decline in offering videoconferencing services across LTCHs post pandemic. Some participants noted shifting funding priorities toward supporting in-person recreational activities rather than diversifying web-based social connection options. In addition, factors pertaining to WVT ease of use and integration included limited staff to support older adults with different physical and cognitive needs, variability in digital literacy including knowledge about accessibility features to enhance the ease of use, and families’ lack of awareness about the availability of WVT for social connectedness.

**Conclusions:**

Web-based videoconferencing technology has the potential to be a meaningful tool to reduce social isolation and promote a sense of social connectedness among older adults and their families and friends. Future research should explore how WVT could be integrated into care planning for this population, particularly in situations where older adults may be at heightened risk for social isolation. Resource allocation toward equipment, infrastructure, and family and staff training would be well-placed to increase engagement with WVT within LTCHs.

## Introduction

### Background

Globally, the COVID-19 pandemic impacts were profound, causing widespread loss of life and morbidity [1,2]. During the earliest phases of the pandemic, significant restrictions were placed on many social activities, including normal activities of daily living (eg, shopping and leisure activities) [3-8]. One specific aspect of daily living that was significantly affected included the notion of *social connectedness* [9,10]. Social connectedness can be described as the subjective sense of being in close-knit relationships with others [11,12]. Having interpersonal relationships and being able to gather with others are integral to individuals’ health and well-being [13,14]. These avenues for social connectedness can prevent people from feeling lonely and reduce the risk of impaired mental health [14-21]. Canadian older adults residing in long-term care homes (LTCHs) were disproportionally affected by the lack of in-person interactions or access to social spaces over the course of the pandemic [22], resulting in their compromised social and emotional well-being [22-24].

During the height of the COVID-19 pandemic, various web-based videoconferencing technologies (WVTs) were introduced by LTCH staff and family members to help maintain social connectivity between older adults residing in LTCHs and their social circles [25-29]. Leveraging WVTs to facilitate social connectivity among older adults was particularly beneficial as these individuals were at greater risk of isolation and loneliness due to factors such as pre-existing isolation (eg, living alone), being reliant on family for support, and impaired physical health such as frailty [30]. Web-based videoconferencing, the use of networked digital telecommunications technology, is a form of telepresence that “simulates the experience of being physically present in a remote environment” [31]. FaceTime (Apple, Inc), Skype (Microsoft Corp), or Zoom (Zoom Communications, Inc) are among the most popular videoconferencing applications used to continue communication among older adults and family or friends when in-person gatherings are restricted [26,32]. Using WVTs enabled people to visually interact with their family members and, in addition to hearing their voice, offered a greater reassurance of their well-being through a visual confirmation [29]. In addition, seeing older adults within LTCHs using WVTs can help foster family members’ belief that their family member was well-cared for [33].

Research suggests that web-based videoconferencing is a viable form of connection for people living in long-term care who may be physically separated from their families and familiar social settings (eg, religious mass services and health care consultations) [34-39]. However, there remains a lack of insight pertaining to the experiences of the individuals directly using this technology. Moreover, additional research is required to inform sustainable and consistent use of WVTs as a reliable option to help older adults maintain social connectedness [40]. Although WVTs offer clear benefits, some literature suggests that this technology may cater more to family members than older adults living in LTCHs, especially if the older adult lives with some degree of cognitive challenge (eg, dementia) [28,33,41]. The types of benefits offered by WVTs across a range of users for the purposes of fostering social connection necessitate further exploration, particularly among older adults with varied cognitive abilities residing in LTCHs. As such, greater knowledge is required to understand how older adults with both physical and cognitive impairments may benefit from or experience challenges in using WVTs [33]. Furthermore, there is a need to understand if and how LTCHs are positioned in terms of infrastructure and staffing to support successful widespread WVT use. For example, arthritis, vision impairments, or neurodegenerative conditions such as Parkinson disease could necessitate the need for adaptive technology or additional assistance as well as greater staff requirements and ultimately cost for the LTCH [42,43]. The potential benefits of WVTs as a tool to support social connection have been shown, but within LTCHs, there is a need to better understand end user experiences if it is to be successfully integrated and sustained as a widespread tool to support social connectedness.

### Objective

The purpose of this study was to understand how older adults living in LTCHs, their families, and LTCH staff members perceived the use and ease of use of WVTs as viable modalities for facilitating social connectedness.

## Methods

### Project Registration

This study presents the findings of the first stage of a multistage qualitative research study described in the published protocol [44].

### Study Design

Using a qualitative description methodology [45], this study sought to explore how older adults in LTCHs, their family members, and LTCH staff experience and understand the use of WVTs to support the social connectedness of older adults in LTCHs. Qualitative description methodology enables researchers to capture and describe a wide range of participants’ experiences and perceptions in a comprehensive, yet clear and practical way using in-depth interviews with study participants [45,46]. In addition, qualitative description is a low-inference approach used in social sciences and health care to provide researchers with a practical way to derive rich, close to data descriptions of participant experiences without the need to interpret the meaning of participant experiences [46]. In addition to in-depth interviews, observational field notes were kept throughout this study, thereby enhancing the dataset and the overall study rigor [47]. Studies using qualitative description methodology seek to find answers that are of high relevance to practitioners or policymakers to improve the quality of a delivered service, including participants’ thoughts or attitudes toward a service; why do participants use certain services more so than others; and how participants use a service in specific instances [45,48].

### Theoretical Framework

The technology acceptance model (TAM) [49] was used to shape interview questions and to organize and report qualitative findings in this study. TAM is a widely used model that helps researchers uncover how end users adopt the use of technological systems and how this adoption is influenced by constructs such as usefulness and perceived ease of use [50]. According to TAM, the actual system use construct concerns the observable use of technology [49] and, in the context of this study, refers to the use of WVTs by the study participants (older adults, family members, and staff in LTCHs) for the purpose of social connection. The perceived usefulness construct is the extent to which technology end users perceive technology as enhancing their tasks or helping them achieve a particular goal [49]. In this study, perceived usefulness is the degree to which participants believe that WVT (devices and software) facilitates social connectedness or makes it more meaningful. The perceived ease of use construct pertains to the amount of effort required to operate the technology by end users [49] and, in this study, reflects participants’ perceptions of how simple it is to operate or navigate WVTs to engage in video calls with friends and family members.

A range of open-ended interview questions was developed using the TAM constructs to guide the in-depth interviews with study participants. For a full list of interview questions, please see Multimedia Appendix 1 in our protocol [44]. In line with qualitative descriptive methodology supporting the use of deductive approaches (ie, TAM) to guide data analysis [51,52], this study presents descriptions of participants’ experiences organized under the following TAM constructs: the (1) *actual system use*, (2) *perceived usefulness,* and (3) *perceived ease of use*.

### The Use of TAM With Qualitative Description Approach

Findings grounded in qualitative description methodology result from staying close to the words and events in the research data capturing straightforward descriptive accounts of participant experiences [45,46]. In some qualitative descriptive studies, findings are categorized under constructs of a chosen theoretical framework [51]. This study incorporated TAM as a framework for organizing data-driven coding and practical and identifiable constructs of the TAM that may be of use to policy makers. The process of incorporating TAM into the data analysis phase ensured that participant data were organized and described in a structured way without imposing subjective interpretations or other theoretical assumptions on the data. Such an approach aligns with previously published research that successfully integrated TAM with qualitative description methodology to describe older adults’ adoption of digital modalities [51,53,54].

### Sample

The desired sample size for qualitative description studies is influenced by factors such as the subject or phenomenon of interest, its representation within the broader population, and the potential variability of experiences with the phenomenon [55]. As such, the recruitment goal of this study was 45 individuals comprised of 3 groups: older adults residing in LTCHs, their family members, and associated staff from 3 participating LTCHs located in southwestern Ontario, Canada. Eligible participants were English-speaking individuals with mild to moderate cognitive disability and able to participate in an interview using the Zoom or Microsoft Teams videoconferencing platforms or a phone. Potential older adult participants with mild cognitive decline living in LTCHs were assessed [56] for suitability to participate in this study by a member of their circle of care within the LTCHs before any study information was shared with them.

### Ethical Considerations

This study was reviewed and approved by the Western University Research Ethics Board (2024-121993-91059). Participants were informed about the research study, and their questions were answered by the study research assistant before they consented to participate. Collected data was anonymized and participants who completed an interview were given a $10 (CAD or $7.20 USD) gift card as an honorarium.

### Recruitment and Data Collection

Participant recruitment and data collection took place between July 2023 and January 2024 using purposeful and maximum variation sampling to help ascertain a sample with a variety of experiences with WVTs, especially among the older adult and family member participants. Research information was distributed to prospective participants via on-premises physical posters, emails, LTCH newsletters, and word of mouth. Individual in-depth interviews with older adults, their family members, and LTCH staff were offered in person, via phone, or by Zoom videoconferencing to accommodate individual preferences and took place at an agreed-upon date and time. Recreational therapy department staff or a family care partner assisted older adult participants in joining the Zoom or interview if necessary. Interviews with participants were audio recorded and transcribed verbatim prior to analysis.

### Data Analysis

The collected data were anonymized and analyzed using NVivo (version 13; Lumivero) qualitative analysis software [57] and directed content analysis, a qualitative analytic technique characterized by a structured stepwise process of coding the data and organizing it into categories and themes [58,59]. The first step of the data analysis entailed deidentification of the cases. Interviews collected from family members were assigned identification such as FM followed by the number of the participant (FM01, FM02, and so on). Similarly, older adults were assigned identification of OA (OA01, OA02, and so on), and staff participants were assigned identification S (S01, S02, and so on). The next step involved reading the interview transcripts, developing memos, and noting down compelling quotes. A formative coding framework was then developed based on the TAM constructs with consensus reached through discussion of the research team. The key constructs of the TAM, perceived usefulness and ease of use of technology, served as initial codes or units of analysis. Data that did not lie within one of the key codes were labeled with a new code and organized into new categories or as subcategories under the initial codes [58]. The preliminary coding framework was tested against the same 5 interview transcripts. Researchers then met on a biweekly basis to refine the coding framework by establishing links between generic and main conceptual categories. The newly identified codes were compared and discussed for similarities and differences until consensus was reached regarding the emergent patterns as the concepts became more densely packed with meaning and evidence. Study rigor was ensured through conducting member checking [60], acknowledging biases researchers may introduce to the research and practicing reflexivity throughout the research processes, and ensuring confirmability of data through triangulation between interviews and field notes [61].

## Results

### Demographic Characteristics Overview

The total sample comprised 39 participants, which is congruent with sample sizes in other studies using qualitative descriptive methods [51]. The sample included older adults (n=7, 18%), family members (n=22, 56%), and staff (n=10, 26%) from 3 participating LTCH facilities, offering diverse perspectives on WVT use in these settings. The 3 participating homes were licensed, not-for-profit LTCHs spanning 120 kilometers and providing comprehensive care to between 160 to 394 individuals, including an emphasis on person-centered care.

All 7 (18%) older participants were female and aged between 64 and 95 years. Older participants were widowed (28/39, 71%) or divorced (11/39, 29%). All 39 older adults either had a college diploma (n=22, 57%) or a university degree (n=16, 42%), providing some context that may be related to the ease of using WVTs. The majority of older adults (n=28, 71%) reported living with cognitive or physical challenges (eg, poststroke impairment, dementia, and arthritis), which provides essential context for understanding the impact of cognitive or physical limitations on WVTs’ usefulness, ease of use, and intention to continue using WVTs. Moreover, 31 out of 39 (nearly 80%) participants in this study identified that physical (eg, arthritis) or mental disability (eg, dementia) affected individuals’ ability to use WVTs.

The 22 family members (FMs) consisted of 18 female and 4 male participants, and were aged between 40 and 69 years. Many family members held a university bachelor’s degree (16/39, 41%), with a notable portion being retired (14/39, 36%).

All 10 staff participants were female, with just under half (10/39, 40%) working in their profession between 6 and 10 years. Staff participants included a range of health professionals such as recreational therapists (12/39, 30%), social workers (8/39, 20%), personal support workers (8/39, 20%), a registered practical nurse (4/39, 10%), and a spiritual care practitioner (4/39, 10%).

### Thematic Presentation of the Findings

#### Overview

Study findings on participants’ roles (older adults, FMs, and staff) and experiences in using WVTs for the purpose of social connectedness are presented in themes informed by the TAM and include the actual system use, with 2 subthemes—(1) WVTs’ hardware and software and (2) trajectory of using WVTs from pre- to postpandemic; perceived usefulness, with 4 subthemes—(1) enabling remote connection, (2) providing emotional and psychological benefits, (3) fostering continuation of life and social roles, and (4) the enriching effect of video presence; and perceived ease of use, with 3 subthemes–(1) design and practicality of the devices and platforms used, (2) usability and accessibility of the devices, and (3) impact on the workload (Textbox 1).

Textbox 1. **Summary of technology acceptance model–informed themes and subthemes.**

**Theme 1: actual system use**

- Web-based videoconferencing technologies’ hardware and software
- Trajectory of using web-based videoconferencing technologies from pre- to postpandemic

**Theme 2: perceived usefulness of web-based videoconferencing technologies**

- Enabling remote connection
- Providing emotional and psychological benefits
- Fostering continuation of life and social roles
- Enriching effect of video presence

**Theme 3: perceived ease of use of web-based videoconferencing technologies**

- Design and practicality of the devices and platforms used
- Usability and accessibility of the devices
- Impact on the workload

#### Theme 1: Actual System Use

##### Overview

The actual system (WVTs) use theme consisted of 2 subthemes: (1) the hardware and software used by older adults, FMs, and staff, and (2) WVTs use trajectory from pre- to postpandemic timeframes.

##### WVTs’ Hardware and Software

During the pandemic, LTCH staff introduced iPads and tablets as a virtual means to facilitate social connectedness between families. Older adults and families also used a range of personal devices with videoconferencing capabilities such as iPads or tablets, laptops, computers, smartboards, and smartphones. Moreover, some families independently researched and purchased nontraditional devices that FMs suggest have more merit for people with physical or cognitive conditions (eg, dementia, arthritis, and paralysis) to access the WVTs they were using, namely, Portal, Alexa Echo Show, and ViewClix hardware. Participants also used various software platforms for WVTs, including Zoom, Teams, WhatsApp, Skype, Facebook Messenger, Signal, and FaceTime (Table 1).

**Table 1.** Web-based videoconferencing technologies used across study participants (N=39).

Web-based videoconferencing technology	Older adults, n	Family members, n	Staff, n	Total, n
Hardware types				
iPad or tablet	6	15	10	31
Computer	3	6	1	10
Laptop	0	4	2	6
Smartboard	0	0	2	2
Smartphone	2	8	3	13
Portal	0	2	0	2
Alexa Echo Show	0	1	0	1
ViewClix	0	1	0	1
Software types				
Zoom	0	10	7	17
Teams	6	1	1	8
WhatsApp	0	1	0	1
Skype	0	3	2	5
Facebook messenger	1	2	2	5
Signal	0	2	0	2
FaceTime	1	12	3	16

##### Trajectory of WVT Use From Pre- to Postpandemic

The iPads or tablets were predominantly leveraged by LTCHs' recreational department teams during the pandemic-related lockdowns and resultant restrictions on in-person visitations as an alternative to socially connect FMs and older adults residing in LTCHs. However, post pandemic, this service was not unanimously integrated into LTCHs’ care delivery. Some LTCHs have embraced the technology, integrating it into daily routines or care planning, while others have shifted funding priorities toward in-person recreational activities, which some FMs shared were not always as meaningful as the ability to see and interact with their loved ones on the screen.

Participants reported varied knowledge of acceptance and normalization of WVT use across different LTCHs from pre- to postpandemic. Most participants who used WVTs to socially connect with people outside LTCHs prepandemic continued to do so during and post pandemic. For these participants, it was the only way to connect with family who lived abroad or far away from LTCHs.

Among the study participants, 14 (36%) participants stated they began using WVTs to support social connectedness due to the pandemic (7 FMs, 5 staff, and 1 older adult). Eleven (28%) participants shared that WVTs continue to be offered by the LTCHs or that they still use it post pandemic (6 FMs, 3 staff, and 1 older adult), while 10 (26%) participants believe the service has stopped being offered post pandemic by the LTCHs (5 FMs, 3 staff, and 1 older adult). A few reasons were provided by the participants to explain the decline in use post pandemic. First, a shift of priorities to do more in-person activities and visitations resulted in a lack of recreational department staff support to help with videoconferencing service. As 1 FM whose mother was admitted to LTCH in March 2023 shared:

*I’ve never had it offered to me with my mom. I don’t think the emphasis is on it and part of it is because the reason they had time to do more of that [videoconferencing] was because so many other programs were cancelled, right?*

[FM21]

A second reason provided was a lack of awareness among older adults and their family members that connecting socially using WVTs was still being offered in the LTCHs:

*If they have anything to offer, they’re certainly not advertising it, I think they don’t have it. I think that it was simply cut.*

[FM19]

A similar concern was echoed by a staff member participant:

*I don’t even know how much recreation [department] even does help with Zoom calls anymore because all family – like anybody can come in now, right? Or they just call too, right? I don’t really see much Zoom, FaceTime, or anything anymore with residents really*.

[S07]

The third identified contributor to the decline of WVT use post pandemic shared by the participants was related to the workload challenges imposed by supporting the use of WVTs to socially connect people:

*We just simply don’t have that manpower to be engaging in this environment as well as supporting you to have a conversation with someone. Even though we understand the value in that, and how important that is for their emotional health, you know, when they are feeling positive and good and they know that their family is safe, they don’t have responsive behaviours*.

[S02]

In summary, the use of WVTs in LTCHs demonstrated benefits for maintaining social connectedness during the pandemic. However, the transition to postpandemic society revealed a decline in its continued use and only partial normalization of videoconferencing across the 3 LTCHs in this study. The trajectory of WVT use from pre- to postpandemic provides insights into the fluid role that technology can have in supporting social connectedness of older adults in long-term care settings.

### Theme 2: Perceived Usefulness of WVTs

#### Overview

The perceived usefulness of WVTs for social connectedness is represented by 4 subthemes: enabling remote connection, providing emotional and psychological benefits, fostering continuation of life and social roles, and the enriching effects of video presence.

#### Enabling Remote Connection

WVTs enabled residents to connect remotely with family and friends, thereby bridging physical distances that in some cases were difficult for FMs to overcome. This opportunity was particularly important for those families who lived at a great geographical distance from the LTCHs or were unable to visit frequently due to various constraints:

*We’ll FaceTime with family members that are far away. I mean, last year, we were basically in four different time zones. My sister was Central, I’m Eastern, my daughter was in the U.K. and one of my nephews was in Japan*.

[FM09]

Conversely, another participant noted a robust example of how WVTs could connect people in the same building:

*At one point [my parents] had to be separated and put on separate floors, so we used a lot of FaceTime and – is it Facebook Messenger? Yes, we were using that between the two of them. So, two of us would have to go in every day and one to my father, one to my mother, so that the two of them could talk*.

[FM03]

In addition, videoconferencing was a useful tool that enabled family to note health care–related details in older adult’s presentation, which they later communicated to the staff, as shared by this FM:

*I recognized when she had an eye infection, or I could see that her hands were really dry. I couldn’t have done that if I was on the phone. We were able to see things and it informed us more about how my mother really was, and it improved her care*.

[FM18]

WVTs were also used for accessing care as an accessible alternative in place of the traditional in-person meetings:

*For the annual team meetings, we had the option to do that virtually or in person. And my sister doesn't drive, and the care home is a long way from her place, so she chose to meet with the care staff virtually, which she really appreciated*.

[FM02]

Those who have not used WVTs for health care access expressed receptiveness to this possibility (FM12). However, those FMs who lived within proximity to the LTCH preferred in-person meetings for such matters. For instance, 1 FM shared that*:*

*there is an annual health care review, and they might have offered that as an option, but I’m just ten minutes from where my mum is, so you know, I would just come in for that meeting.*

[FM16]

Another FM raised an interesting point regarding the nonverbal communication that takes place during the in-person meeting that can be missed or hard to sense over the video call:

*In-person you sense if there’s anything going on. You sense it more because when you are videoconferencing, you may see just one person at a time. But when you’re sitting in the room, we’re all sitting around, and I see everybody, and you feel something if there’s something wrong, if they’re not telling you everything. On videoconferencing you can’t feel it. You just see it, but you don’t feel like there’s any issues*.

[FM22]

These quotes exemplify how WVTs ensured continuous social engagement, helping many residents feel less isolated and more connected to the outside world during lockdown or when they were in isolation.

#### Providing Emotional and Psychological Benefits

Regular videoconferencing interactions helped support older adults’ mental health by reducing feelings of loneliness and isolation. The web-based presence connection with family and friends was a vital source of emotional support. Older adults and their FMs experienced substantial emotional benefits from connecting through the video call via WVTs. It provided them with comfort, joy, and a sense of connectedness, which heightened their positive emotional and psychological well-being:

*The biggest thing it helps with is their mental health, especially if they're just making that change into long-term care. Or if they’ve been in there, to help lift them out of some bad times or bad moments, because I know that depression in long-term care is huge. There’s 24 hours in a day, and if they're sleeping 10 hours and they only get four hours devoted time from someone, there’s a lot of time in between there. So, if there could be some way that they could speak to friends or family or anyone else more, then I think it would decrease depression in long-term care. And that would be big*.

[FM07]

Other examples of the ability of WVTs to provide emotional and psychological benefits include its potential to anecdotally delay the decline associated with a chronic disease such as dementia:

*I think, for the dementia and Alzheimer’s, for her [mom] to visually see the relatives on whatever we use, whether it’s Zoom or FaceTime or Portal, and connect the face with the voice has sort of delayed her [mom’s] decline, because you can actually see them*.

[FM09]

In addition, WVTs were used for prevention of responsive behaviors associated with dementia:

*They [staff] proactively call, because they figure it’ll help settle her [mom] down. So now they do FaceTime because they obviously realize that if she [mom] can see somebody then it calms her down even more than just a telephone call*.

[FM09]

#### Fostering Continuation of Life and Social Roles

Fostering continuation of life and social roles was another subtheme associated with WVT use. For example, WVTs provided an avenue to maintain a sense of normalcy by allowing older adults to continue participating in daily life activities and social roles beyond LTCH walls. For instance, older adults kept in touch with FMs and things that were meaningful to them (eg, their pets), remained updated on family events, and engaged in routine conversations that they would typically have in-person prior to being admitted to LTCHs:

*I was trying to keep [mom] updated, how her pet was doing... show her visually–I would take her for a walk, through the gardens.*

[FM07]

Participants found WVTs useful because they allowed them to fulfill their roles as parents, grandparents, and friends. Even if through the screen, they could still offer advice, participate in family decisions, and stay involved in the lives of their loved ones.

In addition to continuing to fulfill their social roles within the family and household, WVTs allowed older adults to participate in other social events. For example, 1 staff member described how WVTs were used for facilitating music concerts for older adults*:*

*We would get music entertainers at home through video chat and each home area would have on their smart board to get the video chat going.*

[S03]

Another example was using videoconferencing to stream mass in churches facilitated by a spiritual care practitioner (1 staff). As a result, 1 older adult requested to attend a virtual mass daily, as it allowed this resident to feel connected to a church community they belonged to prior to the pandemic. These examples demonstrate the expanded role of WVTs in fostering a sense of connectedness with other community members.

#### Enriching Effects of Video Presence

Compared to other means of communication (eg, landline phone), WVTs were described as having an enriching effect on social connectedness. Participants shared that being able to see faces, share visual experiences, and express emotions more vividly via the screen contributed to a deeper sense of connection:

*How many times you read an email where you take it wrong. How many times you get on a phone, and you don't understand a person may be sounding angry, but they're visually upset. I think the video adds a lot in those situations, particularly since in a nursing home it is so emotional*.

[FM01]

In addition to the interactions between family and older adults, participants described how videoconferencing allowed them to record interactions and watch them later or send them to others (FM01) or observe the resident-staff interactions:

*A side benefit is, every so often I’ll catch one of the staff members in the room with him [my husband], so I have a nice chat with the nurse. I live next door, so I'm there all the time anyway, but it gives me a chance to see the interactions between him and the staff*.

[FM17]

In summary, these findings underscore the multifaceted usefulness of WVTs in maintaining social connectedness and well-being not just for older adults, but also for FMs. Usefulness of WVTs may be hindered or enhanced based on how intuitive and user-friendly these technologies are for older adults, FMs, and LTCH staff.

### Theme 3: Perceived Ease of Use of Web-Based Videoconferencing Technologies

#### Overview

The perceived ease of use was informed by 3 subthemes: design and practicality of the hardware and software, usability and accessibility of the WVTs, and ease of use in relation to the impact on the workload for staff members supporting WVT use.

#### Design and Practicality

Factors impacting the external design of the devices, such as size of the screen, weight, and the availability of supportive accessories like stands, iPad covers, stylus, external speakers, and headphones influenced the practicality and ease of use of WVTs for older adults.

Hardware devices with larger screens, clear audio, and simplified navigation were reported as easier to use. Accessories like headphones or speakers also improved the ease of use. Participants also benefited from using devices with software interfaces requiring minimum to no effort to operate:

*With our mom, she didn’t have to know anything, she just would see faces pop up.*

[FM04]

Portable hardware devices were preferred by some, as they could be conveniently moved and adjusted according to the residents’ needs and preferences and provided more privacy (4 staff). For instance, 1 FM described how they enjoyed going for a walk into the garden and video calling their family from that location. At the same time, portability potentially breached privacy when residents were situated in an open public space like the dining area due to a lack of private space or convenience for staff (1 FM and 2 staff). Eight (21%) participants also expressed their concern for device misplacement, theft, or a risk of accessing passwords and private information through older adults’ personal devices (1 older adult, 5 FMs, and 2 staff), which could be amplified by the devices’ portability.

#### Usability and Accessibility

The usability and accessibility of the devices were key factors impacting participants' use of WVTs. In this study, 31 out of 39 (nearly 80%) participants identified physical (eg, arthritis) or mental disability (eg, dementia) as the primary reason impeding their ease of using WVTs for social connectedness. Both staff and FMs described older adults’ difficulties in comprehending the concept of videoconferencing, which often resulted in disengagement during the video call. Despite this, FMs still appreciated the ability to see their loved ones on screen, even if for a brief period. However, this stimulated FMs to seek other easier-to-use WVTs requiring only the FM to start and end a video call.

In addition to physical or cognitive abilities, digital literacy also varied among participants. Staff noted that the pandemic catalyzed WVT use, and as a result, they had to quickly adapt and engage in learning to use the technologies introduced by their workplaces. Families also engaged in mobile learning either individually or with the help of their technologically literate friends or family. These FMs often assumed responsibility for teaching older adults living in LTCHs how to use WVTs. For those older adults who were particularly isolated or for FMs who lacked support, staff members became responsible for teaching, setting up, or troubleshooting the WVTs for them. One staff member shared a story about teaching a husband outside the LTCH how to download and set up Skype so that he could speak to his isolated wife in the LTCH during the pandemic. Some reported that the assessment for the family and older adult’s capacity to use technology and planning to support the ease of use would usually take place during the resident admission into LTCH (2 staff), although this was not always the case. Findings also suggest that some families were unaware they could set up video calls upon admission (3 family members).

Surprisingly, only 1 FM explicitly received special training on accessibility features embedded into the device through their workplace to enhance its ease of use for people with varied cognitive and physical abilities. Moreover, only a few (3 FMs and 1 staff) were aware of the accessibility features and how to enable them for easier device use.

#### Impact on the Workload

The ease of WVT use impacted the workload among staff within LTCHs. Setting up and supporting older adults with videoconferencing was seen as an additional task to do and required coordination between more than 1 health care team, which led to some resistance to use WVTs among staff. One staff member deployed to assist residents with setting up video calls shared:

*I wouldn’t get the residents ready myself; I would ask a personal support worker to get the resident ready. A lot of times I would go to the floor and some of the residents would still be in bed, so I’d be like: “Someone needs to get this person ready for me because it’s out of my scope*.

[S05]

Another frontline staff member shared that not all staff were willing to facilitate the video calls*:*

*There’s one resident who has her own tablet, who will ask us: “Can you call my daughter?” And we will go and do it for her. Now, when I say we, it’s mostly me and one other person. A lot of people don't want to stop and facilitate that because a lot of times there’s troubleshooting with a tablet. And people aren't always comfortable doing that kind of thing. Or they don't feel like it’s their job to do*.

[S09]

Interestingly, staff reticence to assist with web-based videoconferencing technologies use was received with understanding among family and older adults (1 older adult and 1 FM) with some noting that staff already had many work demands and were too overworked to be helping them with videoconferencing. However, 1 FM also added that contextualizing the reason for a video call (eg, social connection) could overcome the staff’s resistance to help with setting it up and staying with the person for the duration of a video call if needed:

*[…] if you had a palliative resident in the middle of the night that wanted to say last good-byes to somebody, wouldn’t it be wonderful to be able to connect them virtually with somebody that couldn’t be there. I think if it were put in the context like that, it could be woven into some of the other training that you give your nursing staff*.

[FM21]

The perceived ease of use of WVTs in this study was influenced by a range of factors, from choosing the ergonomically suitable device to calibrating it based on unique needs and integrating its use into a care plan given the realities of staff workload.

## Discussion

### Principal Findings

This qualitative study examined perceptions of usefulness and ease of using web-based videoconferencing hardware and software to facilitate social connectedness between older adults living in LTCHs and FMs. In-depth interviews conducted with 3 participant groups—older adults living in LTCHs, FMs, and LTCH staff—suggest that many older adults and FMs benefited from WVTs, and families were creative in finding workable solutions to facilitate older adults’ use of technology despite their potential physical and cognitive limitations. Findings informed by the TAM model were collated in 3 overarching themes: actual system use, perceived usefulness, and perceived ease of use to help inform understanding of WVT use in LTCHs. Key findings in this study include introduction of the WVTs during the COVID-19 pandemic positively impacted emotional well-being of socially isolated older adults in LTCHs and their FMs when in-person visitations were restricted; design and ergonomics greatly influenced the use of WVTs, and staff along with FMs were instrumental in finding ways to make WVTs useful and easy to use, especially for older adults with cognitive or physical impairments; and there was a notable decline in WVT use in LTCHs for social connectedness post COVID-19 pandemic despite heavy reliance on WVT programs to maintain a sense of connectedness during the pandemic.

### Actual System Use

Besides commonly used WVTs like iPads or Zoom platform, FMs and staff participants in this study who provided care to the older adults living with dementia used several devices such as Portal, ViewClix, Alexa Echo Show, and Signal, which are understudied in the literature. For instance, a literature search yielded only 1 study exploring Alexa Echo Show to maintain social connection among older adults without impaired cognitive abilities during the COVID-19 pandemic [62]. Smartphones, iPads, or laptop devices, as well as FaceTime, Skype, or Zoom digital software are the most commonly used WVTs reported in the literature to facilitate social engagement and residents’ emotional well-being [25,29,36,41,63-66]. These hardware and software are useful for many people; however, they might not have embedded accessibility features required for people with special needs, which was noted in this study. In addition, WVT users may require additional assistance, either provided by staff (which may not be realistic to integrate in the current workflow) or due to the variable availability of informal support by friends or family.

Participant experiences highlight the limited number of formal roles (eg, recreational activity specialists), resources (eg, tailored technologies and their maintenance), and programs (eg, digital literacy workshops) to support ongoing WVT use. The lack of WVT use for social connectedness, particularly post pandemic, signals an emphasis on in-person activities and, concomitantly, ongoing staff shortages, which are also reported in other research [67]. The sustained use of WVTs for social connectedness in this study was found among those who were most familiar and comfortable with technology and who were separated by great geographical distances, a finding corroborating other research [66]. However, there were FMs of older adults in this study who were unaware of the ongoing availability of WVTs despite expressed interest in using this modality. This indicates a growing preference for multiple modes of fostering social connection with those residing in LTCHs.

### Perceived Usefulness

Study findings demonstrated that LTCH staff valued using WVTs with families as a means to enhance social presence and for its positive effects on older adults’ well-being, even if they had advanced dementia and were unable to fully comprehend how videoconferencing functioned, which is echoed in other research [36,63,67,68]. Participants in this study and other research [69] reported increased usefulness of WVTs for social connectedness facilitated by LTCH staff or family members, as it allowed older adults to remain remotely connected with their families despite restrictions on in-person visits. Although some of the older adults with more pervasive cognitive shortcomings found it challenging to engage in conversation, videoconferencing experiences still elicited positive reactions and emotions, in accord with findings reported in the literature [36,63,67]. Despite the potential challenges of WVT use, they were perceived as a valuable tool to support well-being, foster engagement between older adults and their family, and enhance patient-centered care [67].

Notable was the lack of clarity voiced by some participants about the availability of and ability to use WVTs, particularly once the acuity of the pandemic had subsided. Although WVT use declined post pandemic in LTCHs, older adults and their FMs described it as a useful and desired service for ongoing use. To achieve optimum quality of life while residing in LTCHs, older adults and their FMs expect greater opportunities for social interactions and higher quality and quantity of family engagement [70], which can be addressed by using WVTs. For example, participants in this study shared that videoconferencing allowed them to stay connected to their life and maintain habits established before moving to LTCHs (eg, virtually visiting the garden they used to walk through and interacting with their pet). This propensity of WVTs to provide access to other contexts may foster a sense of aging in place [71] among older adults who are living in LTCHs.

In addition to supporting social well-being, WVTs may also have an important clinical role, in the form of telemedicine, as these modalities reduce time needed to travel, allow timely access to specialized health care personnel [37] and are a cost-effective solution that could alleviate the need for emergent visits [72]. The added examples of usefulness make it worthwhile not only to sustain but also expand WVT service [73].

### Perceived Ease of Use

Participants in this study independently researched and shared their knowledge of Portal, ViewClix, or Alexa Echo Show. These WVTs were perceived as easier to use for people with cognitive impairment (eg, dementia) than iPads. Although iPads have been commonly leveraged for their convenience and wide societal acceptance, these types of mobile devices often presented challenges for those older adults who had cognitive or physical disabilities or who lacked experience in using WVTs. In line with this finding, higher tablet or iPad use is predicted by factors such as younger age, higher cognitive functioning, and absence of hearing impairment [66]. The use of devices such as Portal, ViewClix, and Alexa Echo Show highlighted in this study warrants further exploration. These devices could be well-situated to address some of the identified challenges of WVT use among older adult populations and in those with increasing health complexity, such as is often found within LTCH settings [74]. This is especially relevant because a lack of social contact was identified as a risk factor for developing dementia, and preventative action is best initiated early [75].

Findings in this study also suggest that staff members noted digital literacy challenges among some FMs as well as older adults living in LTCH, which is echoed across other studies [63,67]. Low digital literacy among older adults may amplify social isolation by excluding these individuals from participating in social processes or spaces [76,77], many of which have shifted their operation to digital spaces in recent years. Moreover, since in many instances the FMs were pivotal for initiating the video calls [36], low digital literacy among them resulted in additional responsibility being assumed by LTCH staff who were required to provide education and manual support [36,67]. These findings highlight the need for broad approaches to foster widespread WVT uses among older adults that consider cost, digital literacy, individual capabilities, usability of these tools, and training and use requirements.

Although WVTs are challenging to use for some older adults [64], looking to the future, more people will likely expect to use and be supported in using WVTs in LTCHs as younger generations of society use these devices regularly and consistently within their daily lives. The importance of digital literacy training and accessibility features to facilitate independent use of WVTs by older adults is documented in the literature [78]. This study also identified limited awareness about, and knowledge of, accessibility features embedded in the WVTs by participants which, in turn, could enhance the ease of their use. Although previous works have identified low digital literacy among older adults [64] and a lack of training available within LTCHs to guide their use of WVTs [36,66], there remains a gap in research regarding implementing digital literacy training programs with older adults in LTCHs and their families. In fact, research suggests that in some cases, FMs have been the ones to take initiative and provide the technology necessary for WVTs within LTCHs [79]. Moving forward, additional funding and education to increase technology availability, enhance digital literacy, and increase staffing support roles within LTCH are warranted.

Staff participants in this study and in others [36,63,67] expressed concern regarding their workload and challenges to integrate videoconferencing with their other roles and responsibilities. Interestingly, a few FM participants in this study were accepting of limited opportunities to connect with their older adult member using WVTs due to staffing shortfalls. It is plausible that widespread awareness of the workload and employment challenges common within LTCHs across Canada contributed to their acceptance [80]. This finding demonstrates families’ awareness and compassion for those employed within LTCHs, but it also highlights the ongoing paucity of resources directed toward supporting the psychosocial well-being of older adults within LTCHs. In addition, it highlights the lack of policy to support widespread use of WVTs in LTCHs. For instance, burdened staff being too busy to provide assistance with WVT use is documented in the literature [66,79]. Institutional supports are paramount to sustain the WVT service for social connectedness and include additional staffing and redefining roles and responsibilities among LTCH staff to foster digital literacy among older adults in LTCHs or, alternatively, to assist older adults and their families in using WVTs [81]. Addressing these factors simultaneously may facilitate smoother integration of this service into LTCHs while limiting the burden on older adults, their families, and staff.

### Implications

Findings from this study highlight the importance of a breadth and depth of approaches to effectively implement and sustain the integration of WVT programs for social connectedness within LTCHs. Government and the private sectors must allocate funding to allow for technology procurement and infrastructure improvements to support wide-scale WVT use within LTCHs. This could be achieved by targeted funding grants or through capital funding initiatives. To maintain WVT use over time, policies and funding to guide its use are also required. Recognition that personnel is required for successful long-term use is paramount if older adults and families are going to benefit. There could be opportunities for expanded roles or even volunteer positions to help older adults use WVTs. In terms of choosing the WVTs, guidelines that ensure accessibility and user-centeredness for individuals with cognitive or physical impairment are required to ensure equitable use of WVTs. In anticipation of the increasingly digitalized health care and potential future increase in using WVTs by individuals in our society, additional roles for staff should be created to be able to support, sustain, and expand WVT programs for social connectedness and health care in LCTHs.

Interdisciplinary care teams in LTCHs can leverage WVTs in their daily routines or activities to improve older adults’ physical and psychosocial well-being and create opportunities for more family engagement. Care teams should create opportunities for older adults and their families to engage in digital literacy workshops to optimize benefits from using WVTs by a greater number of older adults and their families. Staff members should also engage in digital literacy training with a particular focus on troubleshooting WVTs and innovating ways they can incorporate WVTs into their daily activities to promote a sense of meaningful social connectedness among older adults residing in LTCHs.

This study highlights the need to explore long-term impacts on health and well-being of using WVTs with residents and their families. Future studies should focus on exploration and comparison of WVTs to determine the most appropriate WVTs to promote social connectedness, especially for individuals living with cognitive or physical impairments. In addition, metrics on the impact of using WVTs for older adults’ well-being should be collected to determine different aspects of WVTs’ usefulness for older adults in LTCHs. Future research studies should also explore or develop sustainable models to enhance digital literacy training for older adults and their families.

### Strengths and Limitations

A strength of this study is the robust participant sample representing multiple perspectives and experiences in using WVTs for social connectedness. Although staff participants were all female, this parallels research suggesting that 90% of personal support workers employed in Ontario are female and 75% of care workers across Canada are female [82,83]. In addition, collaborating with 3 LTCH sites for participant recruitment supported collection of a diverse range of experiences across the different settings. This mitigated a risk for biased results that would be imposed by the nature and operations of a single facility.

While efforts were made to recruit a range of older participants living in LTCHs, we recognize that the inclusion criteria of mild or no cognitive impairment constrained the participation of older adults. Those who had advanced dementia or other severe neurocognitive deficits were not included, which likely represented a notable portion of those residing in LTCHs who live with dementia [84]. Those older adults who had mild deficits or other physical limitations that impacted their ability to participate were offered assistance in setting up the technology so they could still participate. The findings revealed that frontline staff, particularly nurses and personal support workers, were instrumental in facilitating WVT use among older adults and their families, although this responsibility posed potential burdens on staff workload. Future work could address targeted personnel support for WVT use, including the potential for volunteer technology facilitation or expanding frontline provider roles to include assisting older adults to use technology such as WVTs. Experiences of staff in various LTCH facilities may differ in terms of digital literacy training provided to staff or role expectations. Moving forward, it would be helpful to have clear guidelines to inform staff roles in providing technology support, particularly in the case of supporting older adults’ well-being, including social connectedness. In addition, this study did not delve into other factors that may contribute to the decline in using WVTs post pandemic, like for instance, the complex psychosocial factors that influence technology adoption.

### Conclusions

The experiences of older adults residing in LTCHs, FMs, and staff demonstrate that using WVTs for social connectedness positively impacts older adults and their FMs’ emotional and social well-being. However, WVTs service needs to be tailored to the needs of the families, including the choice of the device, digital literacy training, and provision of human resources to support connections. Moving forward, LTCHs should develop formal programs that allow for integration of WVTs service to expand the opportunities for older adults in the LTCHs to connect socially with their families or use WVTs in other ways that create a sense of social connectedness. To achieve this, funding initiatives such as capital improvement funds and clearer policies on the roles of personnel support will be required. Prospective studies should implement WVTs in collaboration with other actors participating in the process of socially connecting people with WVTs, such as technology industry partners, companies allowing people to participate in or attend leisure activities virtually, and public health organizations to explore additional impacts of WVTs on social and clinical well-being of older adults in LTCHs and their families.

## Abbreviations

FM: family member
LTCH: long-term care home
TAM: technology acceptance model
WVT: web-based videoconferencing technology
